# Directing Experimental Biology: A Case Study in Mitochondrial Biogenesis

**DOI:** 10.1371/journal.pcbi.1000322

**Published:** 2009-03-20

**Authors:** Matthew A. Hibbs, Chad L. Myers, Curtis Huttenhower, David C. Hess, Kai Li, Amy A. Caudy, Olga G. Troyanskaya

**Affiliations:** 1 Lewis-Sigler Institute for Integrative Genomics, Princeton University, Carl Icahn Laboratory, Princeton, New Jersey, United States of America; 2 Department of Computer Science, Princeton University, Princeton, New Jersey, United States of America; 3 Department of Computer Science and Engineering, University of Minnesota, Minneapolis, Minnesota, United States of America; University of Chicago, United States of America

## Abstract

Computational approaches have promised to organize collections of functional genomics data into testable predictions of gene and protein involvement in biological processes and pathways. However, few such predictions have been experimentally validated on a large scale, leaving many bioinformatic methods unproven and underutilized in the biology community. Further, it remains unclear what biological concerns should be taken into account when using computational methods to drive real-world experimental efforts. To investigate these concerns and to establish the utility of computational predictions of gene function, we experimentally tested hundreds of predictions generated from an ensemble of three complementary methods for the process of mitochondrial organization and biogenesis in *Saccharomyces cerevisiae*. The biological data with respect to the mitochondria are presented in a companion manuscript published in *PLoS Genetics* (doi:10.1371/journal.pgen.1000407). Here we analyze and explore the results of this study that are broadly applicable for computationalists applying gene function prediction techniques, including a new experimental comparison with 48 genes representing the genomic background. Our study leads to several conclusions that are important to consider when driving laboratory investigations using computational prediction approaches. While most genes in yeast are already known to participate in at least one biological process, we confirm that genes with known functions can still be strong candidates for annotation of additional gene functions. We find that different analysis techniques and different underlying data can both greatly affect the types of functional predictions produced by computational methods. This diversity allows an ensemble of techniques to substantially broaden the biological scope and breadth of predictions. We also find that performing prediction and validation steps iteratively allows us to more completely characterize a biological area of interest. While this study focused on a specific functional area in yeast, many of these observations may be useful in the contexts of other processes and organisms.

## Author Summary

Genome sequencing has provided us with “parts lists” of genes for many organisms, but many of the biological roles these genes are still unknown. While a great deal of functional genomic data exists, providing information about these genes and their roles, the rate at which these data are leveraged into concrete biological knowledge lags far behind the rate of data generation. Many computational approaches have been developed to generate accurate predictions of gene functions, with the goal of bridging this divide. However, as no large-scale experimental efforts have been based on such approaches, their validity and utility remains unproven. We have performed a study that experimentally evaluates predictions from a combination of three computational function prediction approaches, focusing on mitochondrion-related processes in brewer's yeast as a model system. By using computational predictions to guide our laboratory investigation, we have greatly accelerated the rate at which proteins can be assigned to biological processes. Further, our results demonstrate that in order to achieve the best results, it is important for computational biologists to consider both the underlying data and the algorithmic foundations of the methods used to predict function. Lastly, we demonstrate that iterating through phases of prediction and validation has quickly and extensively expanded our knowledge of mitochondrial biology.

## Introduction

Machine learning and data mining techniques have been applied to a wealth of genome-scale data to produce meaningful predictions of gene/protein involvement in biological processes and pathways [1]–[9]. As biologists have pursued novel findings in a wide range of organisms with finite experimental resources, these approaches have promised to direct experimental efforts toward the most likely targets, with the hope of greatly accelerating the discovery process [10],[11]. However, surprisingly few large-scale experimental studies of gene function have been performed on the basis of computational predictions, despite their great potential to inform and guide such investigations. Perhaps as a result, data continue to be generated at a rate that outpaces the characterization of gene functions [12].

This disparity between the computational and experimental aspects of gene function discovery may be due to a lack of clear demonstrations of the effectiveness of computation in directing laboratory efforts. The few experiments that have been directed by computational systems have generally been limited to confirming individual predictions of the functions of single proteins ([3],[7] and work from our laboratory [13]–[15]). No large-scale studies have been performed to fully explore the ability of computational methods to accurately assign functions to sizeable sets of uncharacterized proteins. Without such comprehensive evaluations, it remains unclear how computational methods can best be employed to guide experimental efforts in discovering novel biology.

To explore the biological considerations important for computational function prediction and to demonstrate the general power of computationally driving experimentation, we have performed a large, systematic study of computational predictions for proteins involved in mitochondrial organization and biogenesis in *S. cerevisiae* [16]. Mitochondrial defects are implicated in a variety of human diseases [17],[18], including neurodegenerative disorders [19],[20] and muscular diseases [21], making them an interesting and relevant target for such a study. The biological mechanisms of mitochondrial biogenesis are largely conserved from yeast through humans (60% of mitochondrial yeast genes have a human ortholog), and as many as one in five mitochondrial proteins are known to be involved in human disease [21],[22]. Mitochondrial biology is understood well enough to provide a sufficient number of training examples for computational prediction methods, but it is also thought that at least a quarter of the proteins involved have not yet been identified [23],[24]. Mitochondrial organization and biogenesis is thus an important and tractable area where computational methods can demonstrate their utility.

In this study, we have examined the biological nature of the predictions made by an ensemble of three computational methods, including supervised and unsupervised techniques that analyze a variety of underlying data. In the companion manuscript [16], we show and describe our biological results using these predictions to direct a suite of experimental tests, including our discovery of 100 additional proteins involved in mitochondrial inheritance. Here, we present detailed analysis of the computational methods and their predictions in order to explore the utility and effectiveness of computational function prediction methods. In particular, we demonstrate several novel observations and conclusions that can greatly impact the use of computational approaches for targeting laboratory experimentation.

First, our results demonstrate that while ∼75% of yeast genes are already known to participate in at least one biological process or pathway, many of these genes may have multiple functions that have not yet been characterized. This refutes the notion of “one gene, one function,” and demonstrates that both characterized and uncharacterized genes are fruitful targets for experimental investigation. Second, by comparison to a new experimental screen of 48 randomly selected genes, we show that using computational predictions to guide laboratory experiments can greatly increase discovery rates. Third, we demonstrate that the specific predictions made by computational approaches are highly dependent on both the algorithmic foundation and underlying biological data employed by those methods. As such, we show that using an ensemble of diverse computational approaches can increase the biological breadth and scope of predictions. Lastly, we demonstrate that by iterating phases of computational prediction and laboratory experimentation, we can greatly expand our knowledge of gene functions.

## Results/Discussion

**Figure 1:**
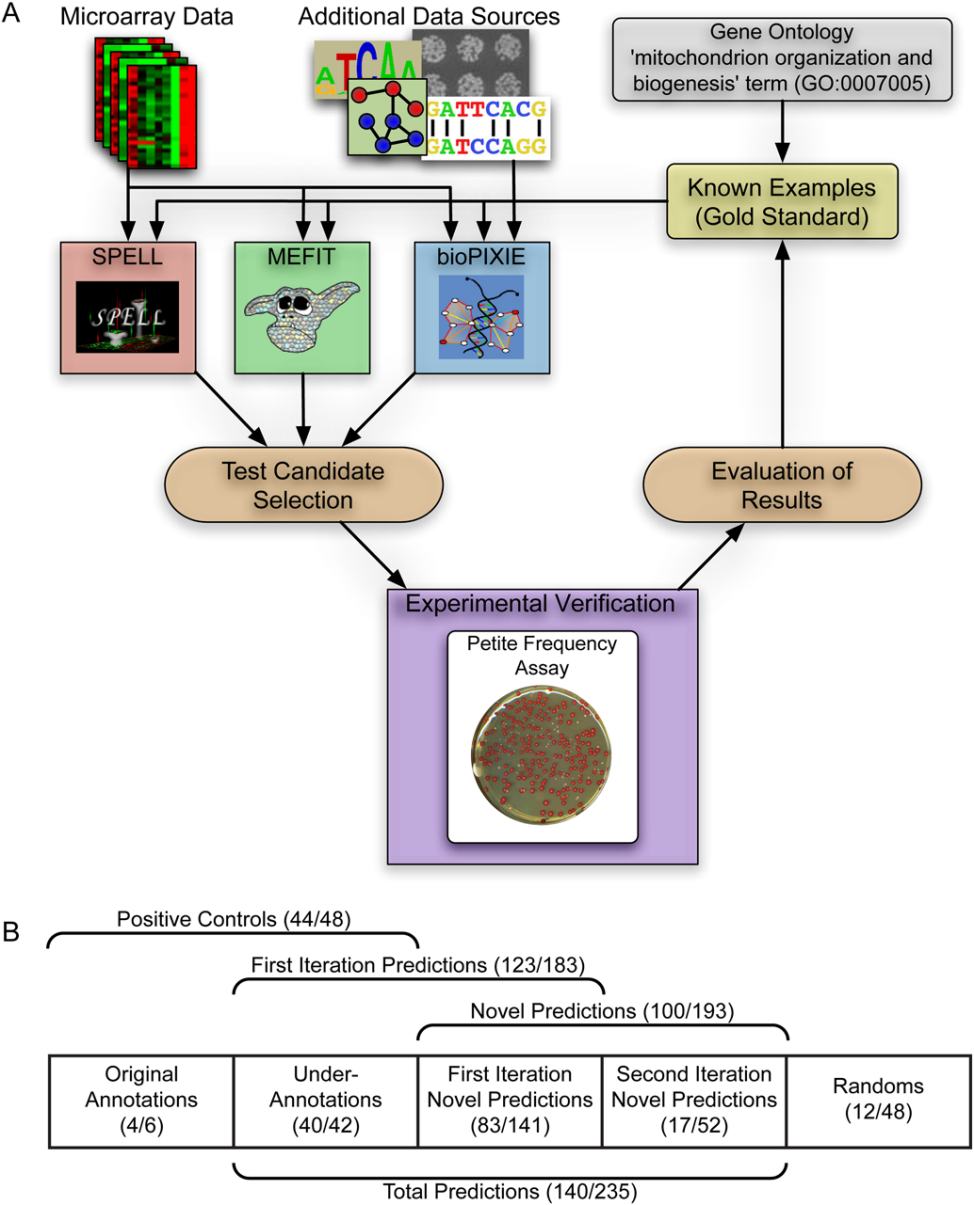
An overview of our iterative approach integrating computational and experimental methodologies. Our study uses an ensemble of computational gene function prediction methods (bioPIXIE, MEFIT, and SPELL) trained and evaluated on known biology to predict novel annotations to the GO term ‘mitochondrial organization and biogenesis.’ (A) The schematic overview of our approach combining computational predictions and laboratory experiments. We selected test candidates based on these computational predictions and validated these novel predictions experimentally using a quantitative, statistically verifiable biological assay. Upon obtaining the results of these tests, the set of known examples was augmented with the validated predictions, and the process was repeated to further explore this biological process. (B) Breakdown of the genes examined at each stage of our study, with the numbers in parenthesis showing the number of verified genes over the number of tested genes. Initially, 183 first iteration prediction genes were selected from our computational ensemble for testing. We found existing literature evidence for involvement in mitochondrial biogenesis for 42 of these genes, and thus included these in the positive control set along with 6 genes that were originally annotated to the ‘mitochondrion organization and biogenesis’ GO term. In our second iteration, we selected an additional 52 candidate genes, none of which had prior literature evidence for involvement. We also selected 48 genes at random from the genome for testing to establish the background genomic rate for our assay.

Our study employed an ensemble of three diverse computational methods (bioPIXIE [15],[25], MEFIT [14], and SPELL [13]) to predict novel genes/proteins involved in the process of mitochondrial organization and biogenesis. Each of these methods integrated high-throughput data sources and utilized existing biological knowledge from the Gene Ontology (GO) [26] and *Saccharomyces* Genome Database (SGD) [27] to identify candidates for involvement. Briefly, bioPIXIE performs context-specific Bayesian integration of a diverse set of genomic data to predict pair-wise functional relationships between genes. MEFIT also performs Bayesian integration, but is targeted to utilize just gene expression microarray data. SPELL uses the same compendium of microarray data, but uses a similarity search algorithm to identify groups of related genes. The results of all three approaches were combined based on the estimated precision of each method to produce our ensemble predictions of gene function (further details are in the Methods section). Predictions for genes involved in mitochondrial organization and biogenesis were validated using a quantitative laboratory assay indicative of involvement in mitochondrial biogenesis and inheritance. The first round of prediction and evaluation used only existing GO annotations as a training set. We then performed a second iteration of this process after updating our training set to include gene predictions confirmed in the first iteration. A schematic view of our system for prediction, verification, and iteration is shown in Figure 1A.

The full biological results of this study are presented in a companion manuscript [16]. The next two paragraphs contain a brief summary of the results important to the additional analyses and conclusions presented here. When the study was undertaken, 106 genes were annotated by SGD to the “mitochondrion organization and biogenesis” GO term (GO:0007005 as of 4/15/2007). These genes were used as input to the computational methods during the first iteration of testing. We initially evaluated our 183 most confident computational predictions, and 123 (67%) were validated as exhibiting a significant phenotype indicative of involvement in mitochondrial biogenesis. Upon further inspection of these confirmed predictions, we found existing literature evidence for 40 of these genes. By following this literature, we found evidence for 2 more of our tested genes and identified an additional 93 genes with strong evidence for mitochondrial function that had not yet been annotated as such by SGD. Many of these genes were annotated to specific categories related to mitochondrial organization (e.g. “integral to mitochondrial membrane”), but were not yet cross-annotated to the “mitochondrion organization and biogenesis” process. In all, we identified a total of 135 genes with existing literature evidence that were “under-annotated.” We have presented this list to SGD and they are evaluating these observations using their established curatorial procedures; as of now, nearly half of these genes have been added to the annotations.

**Figure 2:**
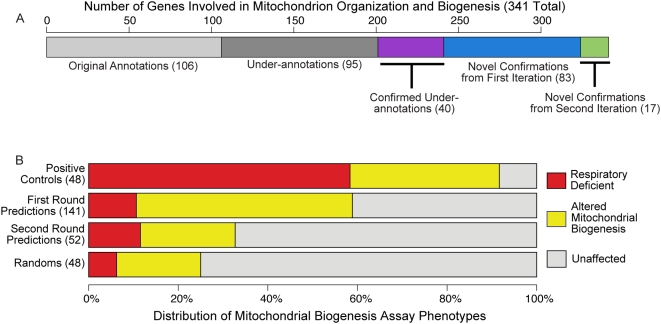
Annotations and phenotypic results for mitochondrion organization and biogenesis. (A) The number of genes involved in ‘mitochondrial organization and biogenesis’ after each stage of this study. Our study began with the 106 genes annotated to the GO term ‘mitochondrion organization and biogenesis.’ In the first round of our iterative computational prediction and laboratory experimentation, we confirmed 123 additional genes. 40 of these confirmations had previously existing literature evidence for involvement in mitochondrial biogenesis, leaving 83 entirely novel discoveries from the first iteration. Based on further literature searches, we found an additional 95 genes with literature evidence for inclusion in this term (including 2 tested genes that did not exhibit a significant phenotype). During our second iteration of testing, we confirmed an additional 17 predictions. (B) The results of our petite frequency assay for genes with previous literature evidence (positive controls), our novel first iteration predictions, novel second iteration predictions, and a random selection of genes. Note that the majority of novel confirmations exhibited the more modest phenotype of “altered mitochondrial inheritance,” whereas the majority of previously known genes are “respiratory deficient,” a more extreme phenotype more easily discovered by high-throughput screens.

Our second iteration of prediction and validation used a set of 324 genes as input to the computational methods (106 original annotations, 83 newly confirmed genes with no prior literature evidence, and 135 “under-annotated” genes). We evaluated the 52 most confident predictions that were not previously tested, and 17 (33%) were validated. While this confirmation rate is still high, the reduction suggests that we may be nearing the edge of genes that can be confidently identified using our assays (details below). Altogether, our study identified 235 new annotations to the process of mitochondrial organization and biogenesis, which more than triples the number of genes previously annotated to this area (Figure 2A). A summary of these results is shown in Figure 1B, and a full catalog of predictions and experimental results is available in Table S1. While these biological results are striking and important, they also have significant ramifications in the application of computational techniques as a whole and in their integration with experimental biology, which we discuss in detail below.

### Many genes with known functions also play additional cellular roles

A common metric for the level of characterization of an organism is the percentage of genes with at least one experimentally confirmed function [10],[12]. By this metric, one might be led to believe that our functional characterization of some model organisms is nearing completion. For instance, in *S. cerevisiae*, we now have established functions for approximately three-fourths of the genome. However, we find evidence that suggests our current understanding is much more limited than these numbers suggest. Among our 193 tested predictions without existing literature evidence for involvement in mitochondrial biogenesis, 75 (39%) are known to be involved in at least one other process, while the remaining 118 (61%) have no previously known function. The verification rate for each of these classes was the same, as 40 of 75 (53%) genes with other known functions and 60 of 118 (51%) genes with no known function were confirmed to be involved in mitochondrial biogenesis. The notion of “one gene, one function” is clearly not consistent with these findings, and we suspect that both uncharacterized genes and genes with previously known functions are fruitful areas for exploration. This issue is even more important when considering higher eukaryotes, where protein variants encoded by the same gene may participate in multiple, diverse functions [28],[29].

Interestingly, there is a strong enrichment for components of the actin cortical patch among the 40 genes newly characterized in mitochondrial biogenesis that also have previously known functions (9 of 40 genes, hypergeometric p<10^−10^). Most genes with known functions specifically related to mitochondrial biogenesis are not included in this number, since they were explicitly reported as “under-annotations” and treated as positive controls for our study. Though the actin cytoskeleton is known to be involved in mitochondrial motility in *S. cerevisiae*, the precise mechanism of attachment and movement has remained elusive [30]. The enrichment of actin cortical patch components is particularly notable since the actin cortical patch has no explicit role in mitochondrial inheritance, but these nine genes are associated with cellular machinery known to move other membrane-bound organelles to daughter cells [31]. Our predictions thus provide evidence that the same machinery may be employed during mitochondrial inheritance in a context similar to, but independent from, their cortical patch roles. By elucidating additional novel functions for previously characterized genes, we not only gain a greater understanding of each protein's individual responsibilities within the cell, we also form a more complete picture of higher-level interactions between cooperating pathways and processes.

**Figure 3:**
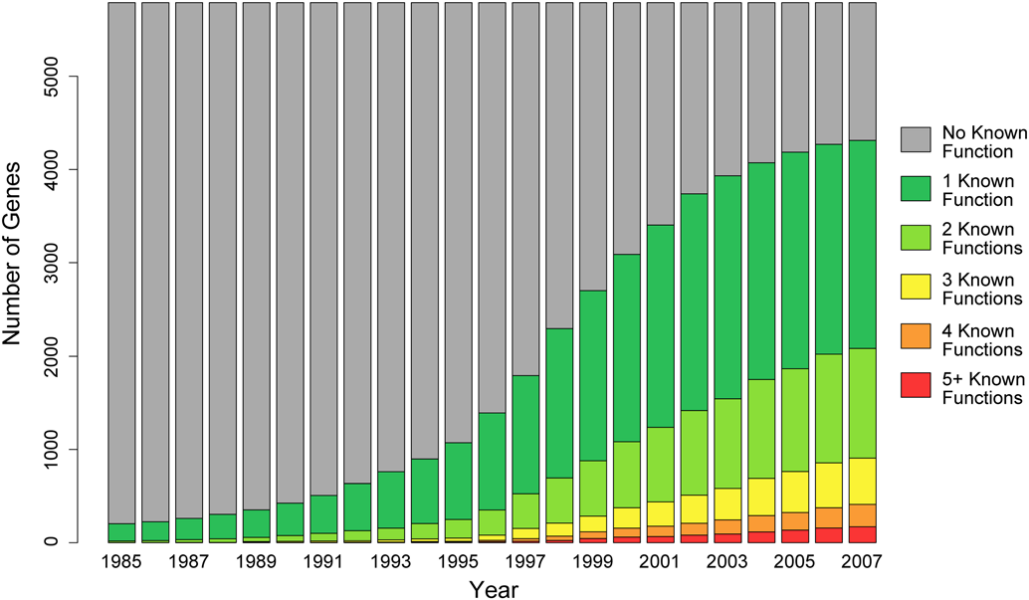
Historical progression of gene function discovery. We examined the historical context of SGD annotations to GO based on the dates of publications used to assign genes to biological processes. Here we define a “known function” as an annotation to a GO term within the GO functional slim mapping [37] for *S. cerevisiae*. Function annotation accelerated after the publication of the yeast genome in 1996, but annotation of multiple functions did not accelerate accordingly.

These results are particularly striking within the historical context of the rates at which gene functions have been characterized. Since the full sequence of *S. cerevisiae* was published in 1996 [32], nearly 3,000 genes have had their first known function characterized, while only ∼1,700 genes have had a second function characterized (Figure 3). It remains unknown how many genes are truly involved in multiple processes, but it is clear that even if single functions were known for all yeast genes, we would still be far from a complete understanding of the complex network that supports most cellular processes. This further underscores the importance of developing approaches for fast and accurate discovery of protein function.

### Guiding laboratory experiments with computation greatly increases discovery rates

Among our 235 experimentally evaluated computational predictions, 140 were verified, resulting in an overall true positive rate of 60%. This result is a striking confirmation that computational predictions can successfully direct laboratory experiments; nearly two out of three predictions were successfully confirmed, which would make even low-throughput follow-up experiments worth pursuing. To quantify our improvement in rate of discovery over the background rate of observing the same phenotypic classes, we chose 48 genes at random to establish baseline rates of phenotypes. Of these 48 genes, only 12 (25%) exhibited a phenotype consistent with involvement in mitochondrial inheritance (data available in Table S1). Based on these results, the use of computational methods to guide our investigation increased our discovery rate by 238%.

In addition to a greatly increased discovery rate, we have evidence that our confirmed computational predictions are more integral to mitochondrial biogenesis than the rare positives resulting from our random screen. As mitochondria are vital for cellular respiration, our assays focused on discovering respiratory defects in single gene knockouts, which is a strong indicator that the tested gene plays a role in mitochondrial processes [33],[34]. However, it is possible for secondary effects of non-mitochondrial mutations to result in similar phenotypes. For example, one of the randomly selected genes tested, *HTA1*, is a histone whose deletion is known to cause pleiotropic effects on transcriptional regulation of carbon metabolism [35]. Consequently, our testing of an *hta1Δ* knockout strain resulted in a phenotype indicating involvement in mitochondrial organization and biogenesis, even though the true cause of this phenotype is likely a secondary effect due to a gross perturbation of carbon metabolism.

Given the possibility that secondary effects could occasionally manifest as positive phenotypes, we cross-referenced our results with known localization information from SGD. We would expect many of the genes involved in mitochondrial organization to localize either to the mitochondrion itself or to the actin cytoskeleton, as mitochondria associate with actin cables for proper inheritance of the organelle during cell division [36]. Among phenotypically positive genes where localization data is available, 72% of our computational predictions are localized either to the mitochondrion or to actin, while only 36% of the 12 phenotypically positive genes from the random screen are similarly localized. The large discrepancy in localization among phenotypic positives from predictions and from the random screen indicates that positive mitochondrial phenotypes in some of the genes in the random screen may be due to secondary effects.

While enrichment for localization to the mitochondria is a strong indicator that our computational predictions are directly involved in mitochondrial maintenance, it is important to note that such localization is not a precondition for involvement. Among all of our novel tested computational predictions, 45% are known to localize to the mitochondrion or actin cytoskeleton, and of these, 59% were confirmed. However, our confirmation accuracy is also high (45%) among the predictions not known to localize to these areas. Thus, if our study examined only genes known to localize to the mitochondrion, it would fail to discover nearly half of the verified genes that resulted from our use of computational predictions. Since computational data integration can leverage a variety of heterogeneous data sources in an unbiased manner, it can successfully direct experimental efforts to targets that might otherwise remain undiscovered.

### Diverse, accurate predictions are made by different computational approaches

**Figure 4:**
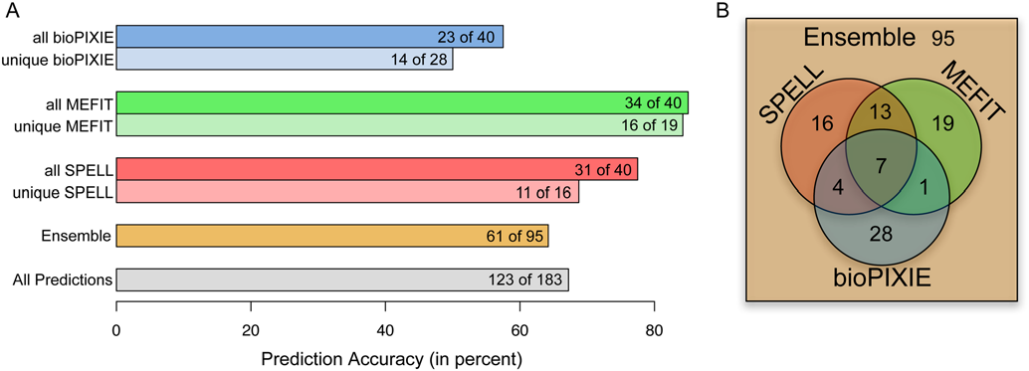
Individual method accuracy and overlap. Three computational methods and an ensemble of those methods were used to select candidates for experimental evaluation. Of the 183 predictions evaluated in our first iteration, 88 were chosen from the top 40 results of at least one individual method, while the remaining 95 were selected from the ensemble of all three. (A) The accuracy of the predictions chosen from each method, from genes selected by the ensemble, and the overall accuracy for all candidates tested in our first iteration. (B) Overlap between candidates selected from the individual methods. Each individual method performs with similar accuracy but predicts unique genes.

In addition to demonstrating the accuracy of computational function prediction approaches, our results also emphasize the importance of considering the specific biological nature of predictions. Specifically, our results show that different computational approaches can produce equally accurate - but distinct - predictions depending on the algorithmic foundation and underlying data of each method. Although we did not attempt a comprehensive study of all types of computational function prediction methods, the three methods used in this study included both supervised and unsupervised approaches utilizing different data sources, and our observations are likely to be generally applicable. To demonstrate this generality, we have also analyzed additional canonical computational function prediction approaches (a Support Vector Machine (SVM) trained using only microarray data, an SVM trained using diverse data, and unsupervised correlation across microarray data). This additional analysis supports the results and conclusions presented below and is fully discussed in Text S1. Each of the three function prediction methods employed in this study achieved similarly high rates of phenotypic positives (Figure 4A). However, there was a relatively small overlap between the 40 most confident predictions of each method, as only 8% of the 88 total candidates selected from an individual method were common to all three (Figure 4B). True positive rates were similar among genes predicted confidently by only one method or by multiple methods, indicating that each computational approach was accurately predicting disparate aspects of mitochondrial organization and biogenesis. This variation can be accounted for both by differences in the underlying data and by algorithmic diversity among the computational approaches. As discussed below, such differences among methods should be carefully considered when developing new prediction techniques or applying them in a biological setting.

#### Underlying data affects the specific biological nature of predictions

Of the three function prediction methods, two are based on detailed analyses of microarray data (MEFIT [14] and SPELL [13]), while the third (bioPIXIE [15],[25]) focuses on integration of heterogeneous data sources such as affinity precipitation results, two-hybrid screens, sequence information, synthetic genetic interactions, etc. As stated, there was relatively little overlap between the three methods' predictions, although all three achieved similar true positive rates during laboratory validation. However, the microarray-based predictions from SPELL and MEFIT did show slightly more correlation with each other than with predictions from bioPIXIE (Figure 4B).

**Figure 5:**
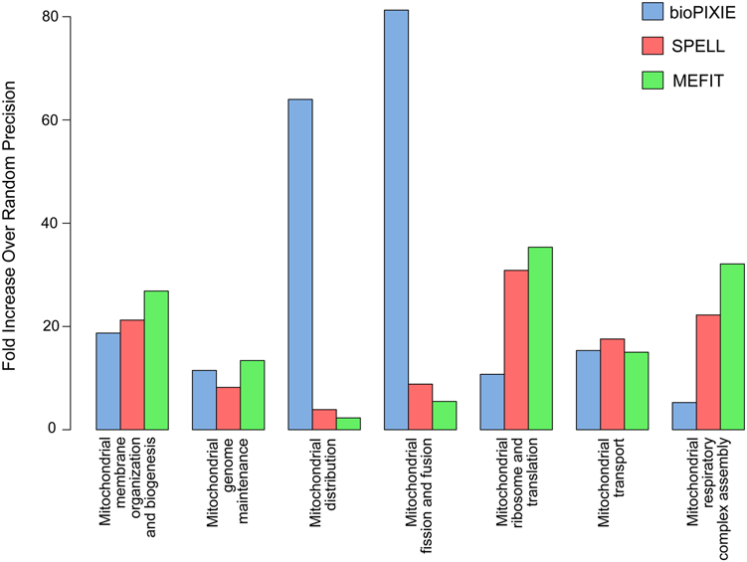
Biological differences between the three computational prediction methods. We evaluated which aspects of mitochondrial biology were targeted by each computational function prediction method. Even though all three methods learned and were evaluated using the same set of training genes, the methods differ in the sub-groups of mitochondrial biology on which they focused. SPELL and MEFIT are both based solely on gene expression microarray data, which explains their strong coverage of the mitochondrial ribosome and translation sub-group. bioPIXIE is based on diverse data, including physical binding data, which explains its strong coverage of sub-groups involving mitochondrial motility and physical interactions.

We characterized the importance of underlying data by examining the cross-validated results for the predictions of each method on more specific sub-processes of mitochondrial organization (Figure 5, see Methods for details). The microarray-based approaches (MEFIT and SPELL) clearly best capture information regarding “mitochondrial ribosome and translation,” which is consistent with other studies that have observed a strong ribosomal bias among microarray data [37]. The method based on diverse data (bioPIXIE) best captured information about “mitochondrial distribution” and “mitochondrial fission and fusion.” This is likely due to the use of physical interaction data, which enables this method to more easily discover proteins involved in mitochondrial structure and motility.

Another significant difference occurs in the area of “mitochondrial respiratory complex assembly,” where the microarray-based methods are more successful than the method based on diverse data. Many of the proteins involved in this process are integral membrane proteins, making them technologically difficult to assay by common sources of physical interaction data (e.g. yeast two-hybrid, affinity precipitation). However, because the number of mitochondria in a cell (and thus the amount of membrane and membrane-bound complexes) depends on environmental conditions, these proteins can be strongly transcriptionally regulated when conditions change. This co-expression is captured by microarray data, providing evidence for our microarray-based predictors of functional relationships.

**Figure 6:**
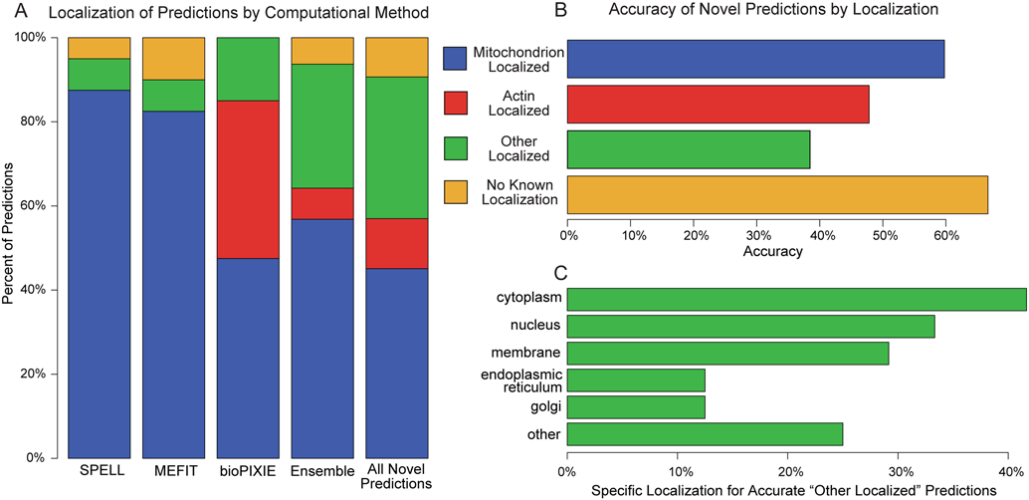
Localization of predictions from computational methods. The known localization of genes predicted by our computational methods differed greatly between the microarray based predictions (SPELL and MEFIT) and the predictions based on diverse data (bioPIXIE). (A) Localization breakdown of the predictions made by each method, by the ensemble, and for all of our novel predictions. (B) Accuracy of our novel predictions by localization. (C) Breakdown of localization for those predictions in areas other than the mitochondrion or actin cytoskeleton. Accurate predictions are not confined to mitochondrion-localized genes, suggesting that computation can discover more diverse gene functions than a screen based only on localization data.

We have also examined the cellular localization of the predictions made by each of the computational methods and those made by the ensemble of all three (Figure 6A). While the majority of the predictions made by the microarray-based methods are known to localize to the mitochondrion, predictions from the method based on diverse data also contained a significant number of proteins known to localize to the actin cytoskeleton. This is consistent with the functional enrichments of the prediction methods, as mitochondria interact with actin for distribution, fission, and fusion. Interestingly, the verification rate was 60% among genes localized to the mitochondrion and 48% for genes localized to actin, but precision was even higher (nearly 70%) among predicted genes with no known localization (Figure 6B). Additional analysis demonstrating the impact of underlying data, including training SVMs with different underlying data is presented in Text S1.

#### Algorithmic differences affect specific computational predictions

Even among methods based on the same underlying data, analyses by different computational approaches can produce very different function predictions. Only 20 of the top 40 predictions made by each of this study's two microarray-based methods (MEFIT and SPELL) overlapped (Figure 4B). However, each method achieved similarly high levels of biological accuracy (Figure 4A), and the functional and localization enrichments of the predictions made by these methods are similar (Figures 5 and 6). These findings can be explained by the fact that these two methods employ very different analytical approaches when generating gene function predictions from microarray data.

One important difference is that MEFIT employs a supervised learning process, while SPELL is unsupervised. MEFIT relies on supervised Bayesian learning to up- or down-weight datasets, using prior knowledge of functional relationships. SPELL performs a query-driven similarity search to identify significant patterns of expression within datasets that are determined to be informative for each query. When performing function prediction, MEFIT infers a complete functional interaction network, which is mined using “guilt by association” for genes predicted to be involved in mitochondrial organization. Conversely, SPELL averages a collection of searches, each querying an individual subset of known mitochondrial genes. (See Text S2 for further discussion of the differences between SPELL and MEFIT. Additional examples of the importance of algorithmic foundations are presented in Text S1.) As the underlying data collection and normalization procedures were the same for both methods, these algorithmic differences account for the diversity of specific predicted genes. This highlights the potential impact of specific algorithms, as well as underlying data, when predicting gene functions.

#### An ensemble of diverse prediction methods increases breadth of results

By employing multiple, complementary functional prediction techniques, we substantially expanded the breadth of our experimentally assayed genes. As described above, the three methods used in this study produced diverse, yet uniformly accurate, predictions spanning many biological aspects of mitochondrial organization and biogenesis. In addition to testing the top 40 predictions of each method individually, we also produced an ensemble prediction set by combining the results of each method based on estimated precision (see Methods for details). From this list, we selected 95 additional candidates for experimental validation.

Thus, approximately half of the novel predictions tested in this study did not occur among the top 40 predictions of any individual method, but were selected based on the ensemble of all three methods. The accuracy of these ensemble predictions is roughly the same (64%) as the predictions made by any of the individual methods (Figure 4). Similarly, the localization and functional enrichments of the ensemble predictions were distinct from those of any one prediction set (Figure 6). By harnessing the diversity and complementarities of our computational prediction methods, we were able to expand the biological scope of our investigation.

### Iterative approaches converge on comprehensive prediction sets

To identify further promising mitochondria-related proteins, we performed a second prediction and validation iteration where confirmed predictions were fed back into the gold standard used in the computational prediction process. Initially, we selected 183 gene candidates to test, 123 of which were verified as likely involved in mitochondrial organization and biogenesis. In addition, we found that 40 of our verified candidates had strong existing support in the literature, which led us to identify 95 further genes with previously published literature evidence for inclusion in this process. After this first round of testing, we created a new training standard of 324 genes including the original annotated genes, the genes with strong literature support, and the experimentally verified genes. Using this updated training set with our ensemble classifier, we selected an additional 52 novel testing candidates, 17 (33%) of which demonstrated a significant phenotype in the lab, resulting in our total of 140 gene function associations (100 entirely novel, 40 with previous literature support). Beyond simply providing additional genes verified to function in mitochondrial biogenesis, this iteration process led us to several important observations.

While the predictions from our second iteration were verified at a rate higher than that of the random set, the discovery rate decreased relative to our first iteration. This suggests that we may be nearing the limit of predictions that can be verified using the single gene knockout assay employed in this study. Among our predictions that remain unconfirmed, some may still be involved in mitochondrial inheritance without exhibiting a significant phenotype using these assays. In our companion manuscript[16], we used our second round predictions to selectively target double-knockouts for many of these remaining predictions with great success.

**Figure 7:**
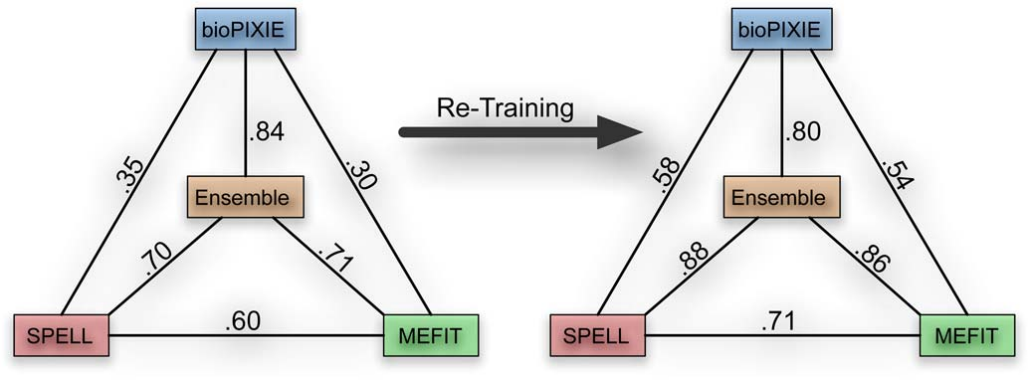
Convergence of computational predictions during iteration. We examined the agreement among our computational methods by calculating the Pearson correlation between the estimated precisions for all predicted genes. After one iteration through our framework, almost all correlations between methods increased; bioPIXIE's correlation with the ensemble decreased slightly due to the ensemble's increased reliance on the other two methods. While the microarray-based methods (SPELL and MEFIT) are most correlated, all methods show significant agreement while providing unique predictions and biological characteristics.

Additionally, while the prediction methods differ with regard to which aspects of mitochondrial biology they best capture (Figures 5 and 6), the methods begin to converge on similar predictions after just one round of re-training. Upon iteration, the correlation between the predictions of each method increased greatly (Figure 7 and Text S1). This convergence indicates that we have expanded our knowledge of this area to a level of biologically reasonable generality, since very different computational approaches can now arrive at similar conclusions. It also suggests that we have successfully avoided bias toward any one functional aspect of the mitochondria caused by over-reliance on individual methods.

These aspects of iterative learning - breadth and convergence - are especially important as the field moves to less well-studied areas of biology and to less well-understood organisms. Iterative applications of computational analysis and directed experimentation provide a means to refine the set of novel predictions and to increase the amount of information used for training. Even when beginning with relatively little information, this process can enable the accurate annotation of a significant number of novel participants in a biological process of interest.

### Conclusion

In order to fulfill the broad promise of computational functional genomics, we must undertake large-scale, iterative efforts to predict, evaluate, and experimentally verify novel gene functions. Our study demonstrates the utility of these types of approaches, and we have made several observations potentially relevant to any computationally directed experimental setting. We find that both characterized and uncharacterized proteins can be fruitful candidates for laboratory investigation. Our results demonstrate that different computational methods can generate accurate but unique predictions, with characteristics dependent on both their underlying data and algorithmic basis. As such, utilizing an ensemble of diverse methods increased the biological breadth of our newly characterized genes. Further, the iterative use of an ensemble with rigorous laboratory experiments allowed us to confirm roles for additional genes and to converge on a refined prediction set.

An important aspect of this study discussed more thoroughly in our companion manuscript [16] is the enrichment of our novel discoveries for subtle phenotypes. Among the novel predictions examined in this study, subtly (but significantly) altered mitochondrial inheritance rates comprised 80% of the confirmed phenotypes; the remainder exhibited the more extreme respiratory deficient phenotype. Of the genes with prior literature evidence, only 36% exhibited altered inheritance rates, while the majority were respiratory deficient. Biologically, this is relevant in the study of the molecular mechanisms of human disease, since genetic disorders are often caused by mutations that only partially impair protein function [38]. From a computational perspective, it represents an opportunity to explore an untapped reservoir of novel biology. Many extreme phenotypes have already been discovered by high-throughput screens. Conversely, experimental assays sensitive and quantitative enough to detect these more subtle phenotypes can be more difficult and time-consuming, and they can thus benefit greatly from computational direction.

Computational methods are critical in a field where the collection of functional genomics data is outpacing the characterization of novel biological knowledge from these experiments. While we used three specific computational approaches to study a particular biological process in yeast, our results demonstrate the broader applicability of combining functional prediction methods with experimental efforts. By directing laboratory investigations to more promising candidates, we can reduce the amount of time and effort required to discover new biology. This includes the characterization of multiple functions for individual proteins, an area still largely unexplored. Through the careful combination and iteration of computational and experimental biology, the rate and breadth of discovery can be enhanced in a variety of conditions, processes, and organisms.

## Methods

A high level overview of our iterative prediction/experimentation/validation approach is shown in Figure 1A. This section briefly details each of the steps involved in this process.

### Computational prediction methodologies

We utilized three complementary computational gene function prediction methods in this study (bioPIXIE [15],[25], MEFIT [14], and SPELL [13]). Each of the methods generated predictions of genes involved in the GO biological process “mitochondrial organization and biogenesis” (GO:0007005). All methods were initially trained and/or evaluated through cross-validation using the 106 annotations to this process as of April 15^th^, 2007. Full details of these methods can be found in their respective publications. Here we present a brief summary of each approach and a description of how each method was used to produce computational function predictions.

bioPIXIE utilizes a suite of context-specific Bayesian networks to predict pair-wise functional relationships between genes, which are then used to create fully-connected graphs weighted by confidence of functional interaction, *w*(*i*, *j*):
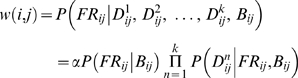
where *FR_ij_* refers to the presence or absence of a functional relationship between proteins *i* and *j*, *D_ij_^n^* refers to the observed association in dataset *n* between the proteins *i* and *j*, *B_ij_* is the biological context of the pair, and *α* is a normalization constant. This method integrates a wide variety of data sources, including physical interaction data (e.g. yeast two-hybrid, affinity precipitation, etc.), genetic interaction data (e.g. synthetic lethality, SLAM, etc.), gene expression data, and sequence data (e.g. coding and regulatory sequence similarity). The Bayesian classifier was trained within the biological process of interest, in this case using the genes annotated to “mitochondrial organization and biogenesis.” Predicted annotations to this term were derived from the resulting weighted interaction network by finding the significance of each gene's connectivity to known mitochondrial genes:
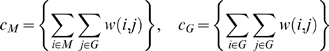


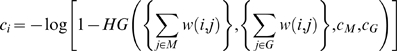
where *c_i_* is gene *i*'s confidence of mitochondrial function, *M* is the set of genes known to be involved in mitochondrial organization, *G* is the set of all genes in the genome, *w*(*i*, *j*) is the predicted probability of functional relationship between genes *i* and *j*, *HG*(*w*, *x*, *y*, *z*) denotes the hypergeometric cumulative distribution function (CDF), and [x] indicates that *x* is rounded to the nearest integer.

MEFIT also predicts pair-wise functional relationships using a GO-trained naïve Bayesian classifier; however, it is based entirely on gene expression data. Both MEFIT and SPELL (below) integrate roughly 2400 microarray conditions which are grouped into ∼120 datasets by publication and further sub-divided by biological process examined. A ranked list of predictions was derived from the mitochondrial organization and biogenesis-specific network by calculating each gene's ratio of connectivity to known mitochondrial genes:
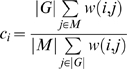
where *c_i_*, *M*, *G*, and *w*(*i*, *j*) are as above.

SPELL utilizes the same gene expression microarray data as MEFIT, but uses a query-driven search algorithm to identify novel players. While SPELL is not trained in a supervised fashion, it assigns a reliability weight to each dataset based on the co-regulation of a specified set of query genes and then orders the rest of the genome based on their weighted co-expression with the query set. SPELL generated predictions by using all possible subset pairs of known mitochondrial organization and biogenesis genes as queries (“leave two in” cross-validation), and then averaged these rank orders together to produce a final prediction list.

Each of these methods generated a ranked list of all genes in order of confidence of involvement in mitochondrial organization and biogenesis. We assigned an estimated precision level (*EP*) to each gene, *g*, in each list by calculating the fraction of genes with a higher confidence level that were already annotated to this GO term (disregarding genes with no biological process annotation or with annotations to the mitochondrial ribosome due to unusually strong expression co-regulation):
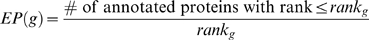



We created an ensemble of the three methods by averaging these estimated precision levels for each gene. In this way each prediction method contributed to the ensemble based on its reliability to recapitulate known biology. Further, this ensemble allows a gene with moderate confidence from multiple methods to rise in the overall rankings.

### Identification of “under-annotated” genes

Our initial evaluation of the computational predictions led us to discover that 40 of our experimentally confirmed predictions were “under-annotated” – meaning that they already had strong literature evidence for their involvement in mitochondrial organization and biogenesis, but were not yet annotated to the corresponding GO term. In most of these cases the information was already curated by SGD in the form of annotations to other GO terms, such as ‘integral to the mitochondrial membrane’ or ‘mitochondrial protein import.’ However, due to the structure of the GO hierarchy, these terms are not directly linked to our process of interest, ‘mitochondrial organization and biogenesis.’ Beginning with these 40 genes, we identified an additional 95 genes that we believe have enough literature evidence to warrant their inclusion in this process without further laboratory testing (including 2 genes tested that did not exhibit a significant phenotype). We have notified SGD of all 135 of these genes, and they are in the process of restructuring the GO hierarchy and making additional annotations. As of submission of this manuscript, SGD has already updated the annotations for more than half of these genes.

### Selection of candidates for experimental testing

Novel candidates for laboratory evaluation were systematically chosen on the basis of both the three individual computational approaches as well as the ensemble of their predictions. As our experimental methodology (described below) is based on assessing phenotypes exhibited by single gene knockout mutants, we limited ourselves to consider only those genes with viable knockouts available in the heterozygous deletion collection. Additionally, we aimed to evaluate both genes with no previously known association to a biological process as well as genes known to be involved in an area other than mitochondrial organization and biogenesis. Thus, we divided the predictions into genes of entirely unknown function and genes with existing biological process annotations.

We selected the 20 most confident genes of unknown function and the 20 most confident genes with existing annotations from each of the three individual methods for testing. Due to overlaps between the methods, this resulted in the selection of 88 genes as novel candidates (the overlap between methods is shown in Figure 4B). We then chose an additional 95 genes from the ensemble list of predictions (38 from genes of unknown function and 57 from genes with known non-mitochondrial function) to arrive at our total of 183 test candidates in our first round of laboratory evaluation. In this way we could evaluate the performance of each individual method as well as the ensemble as a whole.

Of these predictions chosen for testing, we identified 42 as “under-annotated,” whereas the remaining 141 predictions have no previous literature evidence for involvement in mitochondrial maintenance. We selected 6 additional test candidates from the existing annotations to mitochondrial organization and biogenesis, resulting in a total of 48 genes with prior literature evidence for involvement in this process. We also chose 48 genes at random from the set of all viable single gene knockouts in order to establish baseline rates of phenotypic positives. It should also be noted that by chance we would expect some overlap between our random selection of genes and our novel candidates; in our case, 3 genes are in common between these two groups.

### Experimental methodologies and evaluation of results

We utilized a highly quantitative experimental approach to assess a gene's involvement in mitochondrial organization and biogenesis. This method measures a single gene knockout phenotype in comparison to the same phenotype for matched wild type strains. Experiments were performed in replicate for each candidate examined such that robust statistical analysis could be performed on the results.

#### Strain preparation

For all of the genes examined, six independent isolates of complete ORF deletions were obtained from freshly sporulated strains from the yeast heterozygous deletion collection [39],[40].

#### Petite frequency assay

Yeast is able to grow and proliferate even without functional mitochondria on fermentable carbon sources. As such, yeast cells occasionally fail to pass aerobic respiration competent mitochondria on to daughter cells, but these cells can continue to proliferate when a fermentable carbon source is available. Cells lacking functional mitochondria are called petite cells. In this assay we assessed the rate at which single gene knockout strains produced petite offspring. A significantly altered petite formation frequency is indicative of a defect in mitochondrial biogenesis and inheritance [33],[34].

For each mutant strain tested, we grew several replicates of the strain for 48 hours using glycerol as a carbon source. Strains severely deficient in their ability to maintain functional mitochondrial cannot grow on glycerol and were classified as respiration deficient in this first stage. Strains able to grow on glycerol were diluted and plated for single colonies on rich media [41], which releases the requirement for functional mitochondria. Thus, as colonies formed, cells without functional mitochondria were generated. When the colony is fully formed, it is a mixture of cells with functional mitochondria and cells without functional mitochondria. We measured this ratio by re-suspending a colony and plating a dilution of this re-suspension such that 100–300 colonies are formed on a plate. By overlaying with soft agar containing tetrazolium, cells with functional mitochondria were stained red, while cells without functional mitochondria remained white. The ratio of white colonies to total colonies gives the petite frequency. Eight independent petite frequencies were measured for each strain tested. The distribution of these frequencies was compared to the frequency of petite generation in wild-type yeast. Strains identified as having the altered mitochondrial inheritance phenotype in this assay exhibit at least a 20% change in petite frequency from wild type, and have a p-value of less than 0.05 when comparing the petite frequency distributions of that strain to the wild-type petite frequency distribution, using a Mann-Whitney U test.

### Assessing the comparative accuracy of the computational methods

In order to compare which aspect of mitochondrial biology was best captured by each of the computational methods, we created a breakdown of known mitochondrial biology into several sub-groups. Based on the 106 original annotations and the literature evidence for the 135 “under-annotations” we created 7 more specific sub-groups of mitochondrial biogenesis genes shown in Figure 5. Given the prediction ordering of each computational method from our first iteration (i.e. using the original 106 genes as the training set) we calculated the average precision for each of the 7 more specific groups for each of the three computational approaches. The average precision was calculated for each sub-group, *G*, as
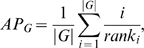
where *rank_i_* is the rank order of the *i^th^* gene appearing from the sub-group in the ordered prediction list. For display in Figure 5, the average precisions were normalized by the expected average precision if the genome were ordered randomly, which corresponds to the number of genes in each sub-group divided by the number of genes in the genome.

### Iterative re-training, prediction, and verification

After our first round of testing, 123 of the 183 predictions were found to have a significant phenotype strongly indicating involvement in mitochondrial organization and biogenesis. Combined with the original 106 annotated genes and the 95 genes identified as “under-annotated,” this results in a total of 324 genes. Each of the three computational methods was re-applied using this updated training set of 324 genes and the same procedure was used to form an updated ensemble list of predictions. We selected the 52 genes with the highest confidence from the updated results that were not previously tested for a second round of laboratory investigation. The same experimental assays and evaluation procedures were used, and an additional 17 genes demonstrated a significant phenotype, resulting in a total of 140 out of 235 total predictions indicating involvement.

## Supporting Information

Text S1 Analysis of additional, canonical computational function prediction methods. Includes Supplemental Figures S1-S3.
(0.68 MB PDF)

Text S2 Algorithmic differences between SPELL and MEFIT affect their specific predictions. Includes Supplemental Figure S4.
(0.17 MB PDF)

Table S1 Summary of computational predictions and results of petite frequency assay.
(0.12 MB XLS)
