# Supplementary material for: Directing Experimental Biology: A Case Study in Mitochondrial Biogenesis
Source: PLoS Comput Biol. 2009 Mar 20;5(3):e1000322. doi: 10.1371/journal.pcbi.1000322 (PMC2654405; doi:10.1371/journal.pcbi.1000322)
Supplement: Text S1 — Analysis of additional, canonical computational function prediction methods. Includes Supplemental Figures S1-S3. (0.68 MB PDF) [file pcbi.1000322.s001.pdf]

Supplemental Text S1. *Analysis of additional, canonical computational function prediction methods*

In addition to the three main computational methods used in this study, we have also performed comparisons with three canonical approaches to function prediction. Our intention with these comparisons is not to claim better or worse overall performance of these approaches (as benchmarking is not the aim of this study), but to demonstrate the generality of the conclusions presented in the main text concerning the importance of underlying data and algorithmic differences when performing gene function prediction.

Description of Additional Methods

We used three additional methods for protein function prediction: a support vector machine (SVM) [1] trained on only microarray data, an SVM trained on diverse data sources, and a normalized microarray correlation (MC) approach.

SVM (microarray data) – We used the SVM Light library[1] and the same microarray data underlying both SPELL[2] and MEFIT[3]. The microarray data was normalized such that all datasets contained log base 2 transformed values and was concatenated into vectors of length ~2000 for each gene. The gold standard positive examples consisted of the original 106 genes annotated to the 'mitochondrion organization and biogenesis' GO term (GO:0007005), and negative examples consisted of genes with a specific annotation at or below the GO functional slim boundary (i.e. terms specific enough to be functionally meaningful)[4] but not to the term of interest. We performed fifty iterations of training and classification, each using a random selection of half of the gold standard examples for training and obtaining classification results for all genes. Final prediction values were determined for each gene by averaging classification results over all iterations where a gene was not included in the training set. These values were sorted to produce the final ordering of predicted genes.

SVM (diverse data) – We applied a similar approach as in the microarray data case, but here we included many additional data types utilized by bioPIXIE[5,6], including physical interaction data, genetic interaction data, localization, etc. Each additional data source was converted into a normalized profile form in a manner appropriate to each data type, and these profiles were concatenated together to form the evidence vectors for each gene.

Microarray Correlation (MC) – For this approach, we examined the Fisher Z-transformed correlations of each gene to the set of 106 genes annotated to the 'mitochondrion organization and biogenesis' GO term (GO:0007005). We used a similar approach as in SPELL, where pairs of genes from the 106 were used as queries and every other gene was ranked by their average correlation to the query pair. All possible pairs were used to create ranked lists, and all of these lists were rank averaged together to produce the final ordering of predicted genes. (Note that this procedure performs the Fisher Z-transformation portion of the SPELL approach, but does not use SVD-based signal balancing nor were datasets weighted by their relevance to the query sets. We also

performed this procedure using raw Pearson correlations across all data, but those results were too poor to make meaningful comparisons with the other approaches.)

Comparison of Results

We selected the top 40 predictions from each of these additional approaches in the same way as for the original approaches (the top 20 genes of unknown function, and the top 20 genes with a known, but non-mitochondrial function). By comparing these predictions between all of the applied methods, we observe the same basic patterns and biases as among the original three methods. We show the overlaps between these approaches and the original approaches in Supplementary Figure S1.

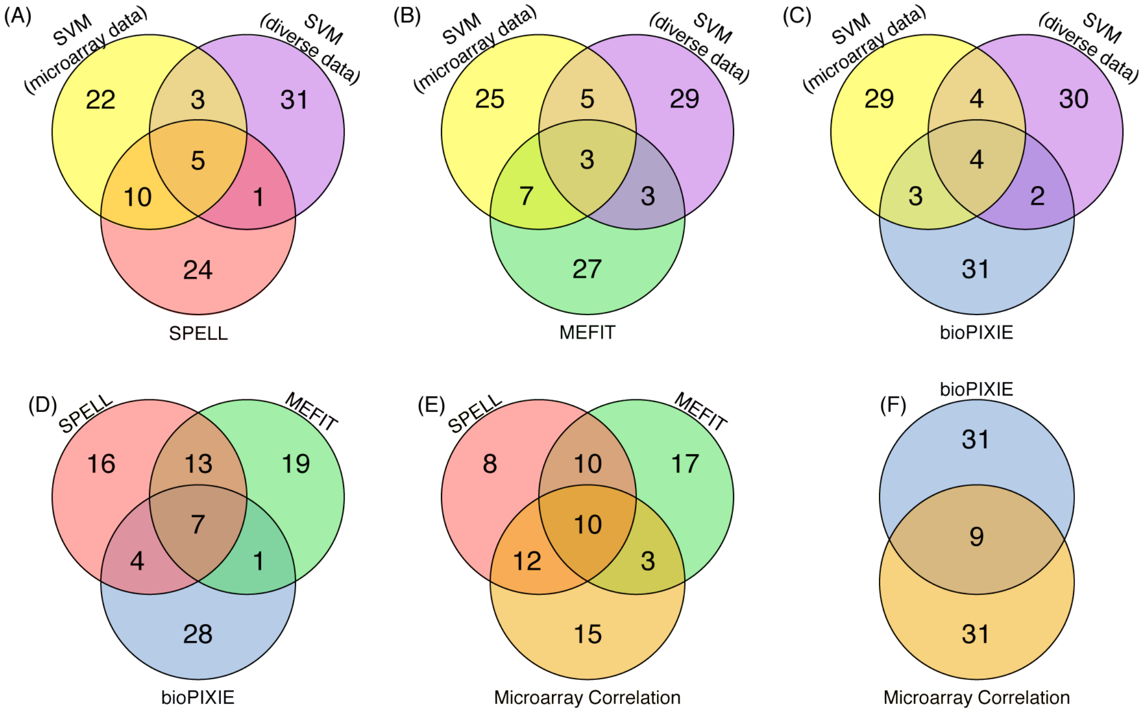

Supplementary Figure S1 – Overlap between the top 40 predictions made by additional, canonical methods and the three methods used in the main study. (A-C) shows comparisons between the SVD based methods and the three main methods. (D) shows the overlap among the main methods, and is reproduced from Figure 4B. (E-F) shows comparisons between Microarray Correlation (MC) and the three main methods.

Importance of Underlying Data

Just as the microarray-based methods SPELL and MEFIT agreed more with each other than with bioPIXIE, which is based on diverse underlying data, the SVM trained using microarray data agrees more with SPELL and MEFIT than does the SVM trained using diverse data (see Fig S1A-B). Also, using simple microarray correlation (MC) agrees better with SPELL and MEFIT than bioPIXIE (see Fig S1E-F). This agreement also extends to some of the specific functional breakdowns of these predictions, as shown in Supplmentary Figure S2. The SVM based on microarray data, the MC approach, MEFIT, and SPELL all perform very well for the subset of mitochondrial biogenesis

genes related to the mitochondrial ribosome and translation, which is an area where microarray data has been shown to perform well[4]. Additionally, the microarray-based SVM performed well for the sub-function of mitochondrial respiratory complex assembly (as did MC to a lesser extent), which agrees with our observation in the main text that microarray data may contain more information about this sub-function than many other data sources, such as physical interaction data. Further, as shown in Supplementary Figure S3, the top predictions of all four of these microarray-based methods are largely localized to the mitochondrion, while the methods based on diverse data made fewer predictions similarly localized. This difference is consistent with the observation that microarray data contains information regarding mitochondrion-localized complexes involved in translation and respiration.

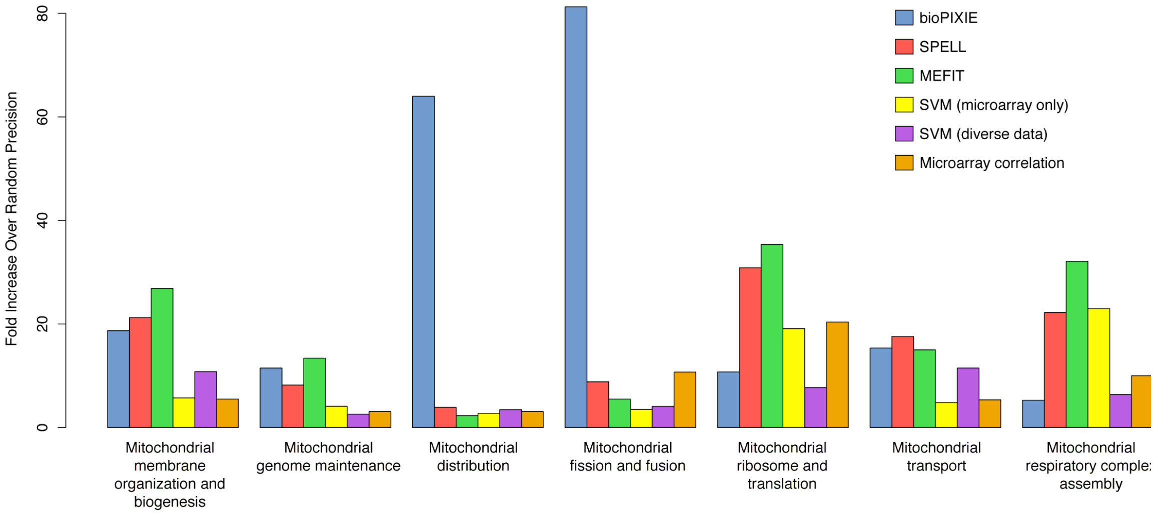

Supplementary Figure S2 – Performance of the three main methods and three additional methods on sub-functions of mitochondrial organization and biogenesis. This figure is analogous to Figure 5 in the main text and was generated in the same manner.

Importance of Algorithmic Foundations

Interestingly, we also observe large differences between the diverse data based bioPIXIE and the SVM trained using diverse underlying data (see Fig S1C). We can understand this difference by examining the functional and localization breakdowns of predictions made by all of the methods (see Fig S2 and S3). These analyses show that unlike bioPIXIE, the SVM trained on diverse data does not make any actin-localized predictions and does not perform well for the sub-functions of 'mitochondrial distribution' or 'mitochondrial fission and fusion.' This difference is most likely due to the different algorithmic foundations of these two approaches. In short, bioPIXIE's algorithm utilizes a pair-wise graph of predicted gene interactions to make predictions, while the SVM approach attempts to find the best binary classification of genes. This can greatly affect predictions when subsets of genes are strongly related within, but not between, these subsets. In this case, of the 106 genes used as positive examples to train these methods, only 11 (10%) are localized to the actin cytoskeleton and only 9 (8%) are known to be involved in mitochondrial fission and fusion. However, in the pair-wise network generated by bioPIXIE these groups are very heavily connected to each other and to

additional candidate predictions. This causes bioPIXIE to perform well for these groups and produce many predictions related to the interaction between mitochondrion and the actin cytoskeleton required for proper mitochondrial motility. Conversely, the SVM approach is likely to overlook these strong, but infrequent, relationships in favor of finding other genes related more generally to the entire 106 genes used for training.

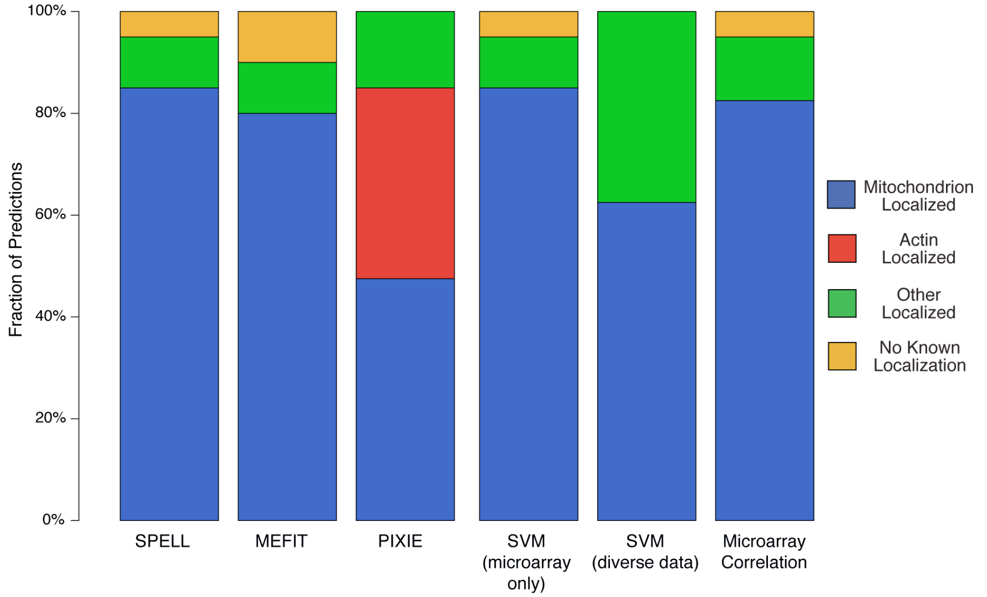

Supplementary Figure S3 – Localization of the top 40 predictions made by the main three methods and three additional methods. This figure is analogous to Figure 6A in the main text and was generated in the same manner.

Discussion

The prediction results from an SVM based on microarray data, an SVM based on diverse data, and simple microarray correlation (MC) generated predictions with characteristics consistent with our conclusions in the main text regarding the importance of underlying data and algorithmic foundations. This further strengthens our observation that the functional aspects of generated predictions must be considered in addition to their accuracy in order to make meaningful comparisons between computational function prediction approaches. While we did not experimentally test the unique predictions among the top 40 produced by the SVMs or MC approach, the accuracy of the predictions that overlapped with the approaches used in the main study was between 65-73%. This is very comparable with the accuracy of the overlapping predictions in the top 40 of MEFIT, SPELL, and bioPIXIE, which was 75%. As such, we could reasonably expect that a number of the unique predictions produced by the SVMs and MC approach to be accurate. Thus, just as using an the ensemble of three methods in the main study broadened the biological scope of the predictions examined, we could potentially further improve our ensemble by incorporating additional function prediction techniques.

References

1 Vapnik VN (2000) The Nature of Statistical Learning Theory. Springer.

2 Hibbs MA, Hess DC, Myers CL, Huttenhower C, Li K, Troyanskaya OG (2007) Exploring the functional landscape of gene expression: directed search of large microarray compendia. Bioinformatics 23: 2692-2699.

3 Huttenhower C, Hibbs M, Myers C, Troyanskaya OG (2006) A scalable method for integration and functional analysis of multiple microarray data sets. Bioinformatics 22: 2890-2897.

4 Myers CL, Barrett DR, Hibbs MA, Huttenhower C, Troyanskaya OG (2006) Finding function: evaluation methods for functional genomic data. BMC Genomics 7: 187.

5 Myers CL, Robson D, Wible A, Hibbs MA, Chiriac C, et al (2005) Discovery of biological networks from diverse functional genomic data. Genome Biol 6: R114.

6 Myers CL, Troyanskaya OG (2007) Context-sensitive data integration and prediction of biological networks. Bioinformatics 23: 2322-2330.
