# Supplementary material for: Directing Experimental Biology: A Case Study in Mitochondrial Biogenesis
Source: PLoS Comput Biol. 2009 Mar 20;5(3):e1000322. doi: 10.1371/journal.pcbi.1000322 (PMC2654405; doi:10.1371/journal.pcbi.1000322)
Supplement: Text S2 — Algorithmic differences between SPELL and MEFIT affect their specific predictions. Includes Supplemental Figure S4. (0.17 MB PDF) [file pcbi.1000322.s002.pdf]

Supplemental Text S2. *Algorithmic differences between SPELL and MEFIT affect their specific predictions.*

As discussed in the main text, even among methods based on the same underlying data, very different predictions can be made depending on the computational approach used to analyze the data. We observed significant differences between the two microarray-based methods used in this study (SPELL and MEFIT), even though they are based on the same input data and each method achieved similarly high levels of biological accuracy (Figure 4).

There are several potential reasons for algorithms utilizing the same data to generate disparate predictions. Data normalization, distance metrics, training sets, evaluation metrics, algorithm type, and parameter choices can greatly affect the predictions generated by a computational approach. For this study we used the same set of ~120 microarray datasets initially normalized in the same manner, using the same distance metric, and evaluated on the same training set. Despite these similarities, there are significant differences between these two approaches that affect their predictions, as outlined below. The full details of these methods can be found in their respective original publications [1,2].

Brief Algorithmic Descriptions

MEFIT trains a naïve Bayesian network in a supervised fashion using known examples of genes involved in a specific biological context (in this case, mitochondrial organization and biogenesis). Fisher Z-transformed, z-scored gene correlations are binned into 8 ranges for use in the network. The training process results in a conditional probability table for every microarray dataset, which contains the probabilities of observing each bin of correlation between functionally related and un-related pairs of genes. MEFIT then performs inference on the trained network to predict the likelihood of pair-wise functional relationships between all genes to form a fully connected, weighted functional interaction network.

MEFIT generates predictions of gene function by examining connectivity properties of the resulting weighted functional interaction network. MEFIT ranks genes by the ratio of their connectivity to known mitochondrial organization genes over their connectivity to all genes in the genome. Thus, genes that are strongly connected to known mitochondrial organization genes are the most confident predictions.

SPELL uses a query-driven, unsupervised similarity search to identify functionally related genes. Here, similarity is defined as the Fisher Z-transformed, z-scored correlation in an SVD signal balanced projection of the original data, where only positive correlations are considered. Given a set of query genes, SPELL assigns a weight to each dataset proportional to the similarity among all of the query genes within each dataset. Then for every other gene, a weighted correlation score is calculated such that genes with expression patterns similar to the query set in datasets where the query genes agree

receive the highest score. The entire genome is ranked by this score, and the ordered results are returned.

SPELL generates predictions by executing a series of queries, each using a subset of known mitochondrial organization genes, and averages the results together to form a final ranked order. For this study, all possible pairs of mitochondrial organization genes were used as queries, and the resulting prediction orders were averaged together by rank to produce the prediction list.

Some Important Differences

- MEFIT's use of binned correlations is quite different from SPELL's use of continuous positive correlation. MEFIT is able to capture signals present among anti-correlated gene pairs, while SPELL is limited to considering only positive correlations. However, SPELL does not bin data and therefore can capture more fidelity among correlations, but may also be more susceptible to noise.

- While both methods consume the same input data normalized in the same manner, SPELL calculates correlations in a re-projection of the original data. This projection is based on Singular Value Decomposition (SVD) and is designed to dampen the dominant signals and amplify more subtle signals in the original data.

- MEFIT selects prediction candidates based on their global connectivity to all known mitochondrial organization genes, whereas SPELL rank-averages a series of local searches to identify candidates.

- MEFIT utilizes a negative training set of genes known to not participate in mitochondrial organization, while SPELL utilizes only the query genes provided. Thus, MEFIT is better able to handle cases where a level of correlation is uninformative as it occurs in both positive and negative training examples.

Discussion

While there are significant differences between these two methods that account for the diversity among their predictions, there are also important similarities. Each method essentially assigns a reliability weight to each dataset in the compendium (from MEFIT's conditional probability tables, and SPELL's query set similarities). While the dataset reliability weights obtained by each method differ, we found that they were significantly correlated ($r=0.55$, p-value$=6\times10^{-10}$, see Supplementary Figure S4). Thus, each method is able to locate and utilize some overlapping signal present in the original data. Further, just as the method predictions converged during the retraining process, the dataset weights of these methods also converged after retraining ($r=0.71$, p-value$=2\times10^{-18}$).

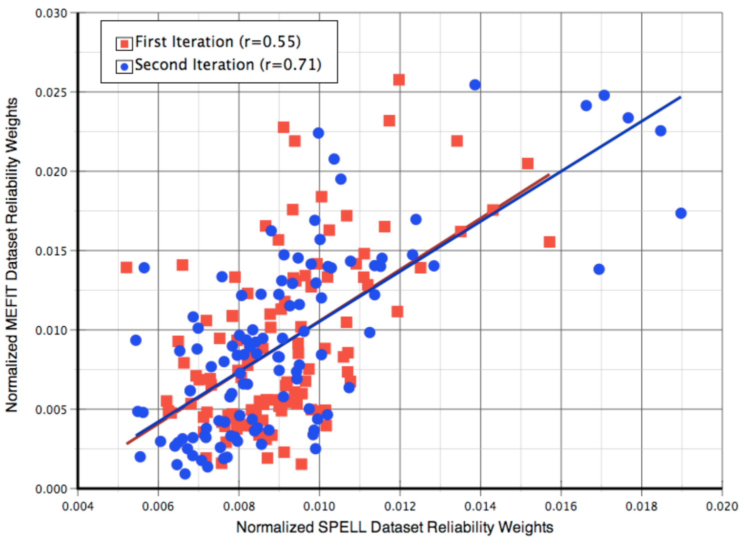

Supplementary Figure S4 – Correlations between microarray dataset weights used by SPELL and MEFIT during the first and second iterations of testing.

References

1 Hibbs MA, Hess DC, Myers CL, Huttenhower C, Li K, Troyanskaya OG (2007) Exploring the functional landscape of gene expression: directed search of large microarray compendia. Bioinformatics 23: 2692-2699.

2 Huttenhower C, Hibbs M, Myers C, Troyanskaya OG (2006) A scalable method for integration and functional analysis of multiple microarray data sets. Bioinformatics 22: 2890-2897.
